# Theories and Quantification of Thymic Selection

**DOI:** 10.3389/fimmu.2014.00013

**Published:** 2014-02-04

**Authors:** Andrew J. Yates

**Affiliations:** ^1^Departments of Systems and Computational Biology, Microbiology and Immunology, Albert Einstein College of Medicine, New York, NY, USA

**Keywords:** thymic selection, T cells, mathematical modeling, repertoire selection, theoretical biology

## Abstract

The peripheral T cell repertoire is sculpted from prototypic T cells in the thymus bearing randomly generated T cell receptors (TCR) and by a series of developmental and selection steps that remove cells that are unresponsive or overly reactive to self-peptide–MHC complexes. The challenge of understanding how the kinetics of T cell development and the statistics of the selection processes combine to provide a diverse but self-tolerant T cell repertoire has invited quantitative modeling approaches, which are reviewed here.

## Introduction

Conventional (CD4^+^ and CD8^+^) T cells are an integral part of adaptive immune systems in vertebrates. A key stage in their development is the creation of the T cell receptor (TCR) through a stochastic process of gene rearrangement. The resulting pre-selection TCR repertoire has the potential to recognize a very large array of peptides derived both from self and from foreign organisms, presented on Major Histocompatibility Complex (MHC) molecules on the surfaces of other cells. Much of T cell development occurs in a specialized organ in the chest called the thymus, within which this diverse potential repertoire of TCR is vetted. A process referred to as positive selection removes cells with TCR conformations that are generally non-responsive to self-peptide–MHC ligands (self-pMHC), and negative selection removes cells that are overly reactive to self-pMHC and pose a threat of autoimmune responses. The post-selection repertoire exported from the thymus comprises T cells that are largely non-responsive to self, yet capable of responding with remarkable specificity to foreign peptides.

**Figure 1:**
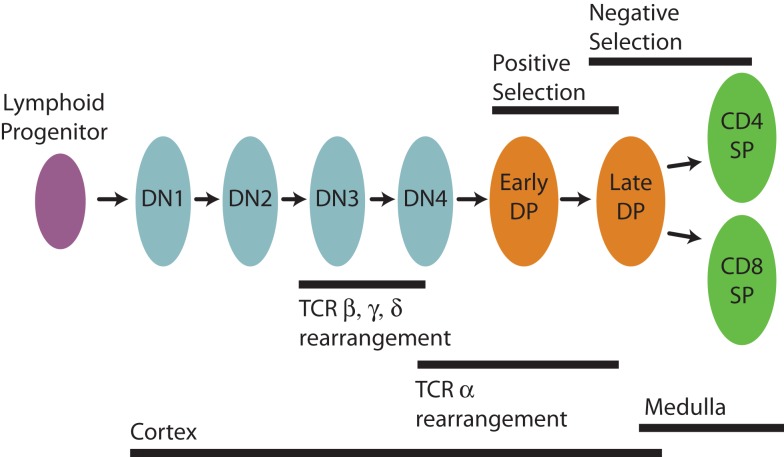
**Stages in the development of CD4 and CD8 T cells in the thymus**.

There is a very extensive literature relating to thymic development and selection [for reviews, see for example Ref. (1–3)], but here we summarize the key ideas briefly (Figure 1). Conventional T cells begin life as lymphoid progenitors, which migrate from the bone marrow to the inner, cortical region of the thymus and begin a process of proliferation and maturation. Early in development in the cortex thymocytes are referred to as double negative (DN), lacking expression of the CD4 and CD8 co-receptors that are involved in TCR signaling. The TCR comprises two chains and is formed by a multi-step gene rearrangement process that first generates the TCR*β, γ*, and *δ* chains (a small proportion of cells diverge at this stage to seed the *γδ* T cell lineage) and then the TCRα chain at around the transition from the DN to CD4^+^CD8^+^ (double positive, DP) stage. TCR*αβ* cells then migrate among cortical thymic epithelial cells and dendritic cells, auditioning for the ability to recognize self-pMHC. There is evidence that DP cells with non-functional TCR can undergo repeated TCR*α* rearrangements (4) to re-audition. Positively-selected cortical thymocytes begin negative selection and eventually move to the outer capsule of the thymus, the medulla. There they complete negative selection through interactions with medullary thymic epithelial cells and dendritic cells. TCRαβ thymocytes, which recognize self-peptides presented on MHC class I or class II below an acceptable threshold of reactivity develop into the CD8 SP (single-positive, CD4^−^CD8^+^) or CD4 SP (CD4^+^CD8^−^) lineages, respectively, and are eventually exported into the peripheral circulation as naive T cells.

The topic of thymic selection has received substantial attention from the immunological modeling community, perhaps for two main reasons. First, selection has widely been viewed as a well-delineated optimization problem – how to craft a TCR repertoire that covers the space of possible pMHC ligands as widely as possible, while preserving sufficient specificity to discriminate between self and foreign (and between different foreign) peptides? This question naturally invites quantitative arguments. Second, the biology is well-characterized – a relatively small number of cell types and modes of interaction appear to be involved, and large amounts of experimental data are available. These simplify and constrain the construction of models.

Modeling studies have focused on many aspects of thymic selection but many questions and uncertainties remain. What are the rates and efficiencies of passage through the different phases of development and selection, and in what thymic microenvironments do each take place? How do thymocytes integrate signals received from interactions with pMHC to make fate decisions? What are the relative contributions of the MHC itself and its associated peptide to TCR signaling and fate determination? What influence do each of these have on the post-selection repertoire’s diversity and coverage of the pMHC universe, and its ability to discriminate between self and foreign? How complete is the removal of potentially self-reactive clones? How many TCR interactions contribute to a thymocyte’s fate decisions? What evolutionary pressures have determined the typical number of MHC alleles we possess? There have been many different theoretical approaches to these questions – from mean-field population dynamic models of progression through developmental stages, to probabilistic models of selection, to explicitly spatial models of migration within the thymus.

This review groups studies of these topics into broadly labeled categories, but in some cases the grouping is arbitrary – many of these questions are related and have been addressed either alone or in combination. The review has a bottom-up structure, beginning with an overview of experimental quantification of selection and modeling of thymocyte population dynamics. It then moves to studies of higher-level properties of the T cell repertoire, such as TCR cross-reactivity, and concludes with the problem of optimal within-individual MHC diversity.

## The Population Dynamics of Thymocytes

Basic elements of a quantitative understanding of thymic development are the steady-state population sizes of different developmental stages, the mean times to transit between them and the proportion surviving at each stage, which we refer to as the efficiencies of selection. While some quantities can be experimentally determined, mathematical models have helped us develop a more complete description of the kinetics of selection, both for the thymocyte population as a whole and for the CD4 and CD8 lineages in isolation.

To estimate the parameters of a dynamical system usually involves observing its response to perturbations. One method is to follow cohorts of cells as they progress through development using intra-thymic injection of a dye or radioisotope label (5–8). Arguably this method is less disruptive than cell transfers, but the uptake of marker can be heterogeneous (5, 7) and measurements of death rates using injected dyes rather than congenic markers may be confounded by loss of label (9). More recently, methods have included using GFP (green fluorescent protein) expressed during TCR rearrangement, its decaying intensity then a marker for time spent in development (10); inducible TCR signaling can be used to arrest, release, and follow cohorts of cells from the early DP stage (11); and small numbers of labeled thymocytes isolated at different developmental states can be followed after intra-thymic injection (11, 12). The population dynamics have also been exposed by transiently depleting thymocytes and observing the system’s return to equilibrium (13). Various experimental systems, with or without associated dynamical models, are in general agreement over several quantitative aspects of thymic development but inconsistencies and uncertainties remain.

### Selection efficiencies and cell fluxes

Thymocytes begin to select against self-pMHC ligands at the DP stage following TCR rearrangement and so we focus on survival, proliferation, and differentiation from this stage onward. The proportion of DP cells that reach maturity (that is, survive both positive and negative selection) is widely agreed to be 5% or less (6, 11, 13–16). Within this pruning process, the general view is that positive selection is the most stringent, with 75–80% of cells failing to progress from the earliest DP stage, suggesting the majority of TCR generated are unable to recognize peptides in conjunction with MHC class I or II to any useful degree (11, 13, 15, 17, 18). Many studies have estimated that between 20 and 50% of positively-selected thymocytes then survive negative selection (11, 17, 19–22), although Itano and Robey (8) estimated a selection efficiency as high as 90% for DP cells into the CD4 SP lineage.

The rate of production of mature CD4 and CD8 cells in the thymi of young adult mice is roughly 1% of total thymocytes or 1 − 3 × 10^6^ cells/day, a figure arrived at by a variety of labeling methods (5–7). Egerton et al. (6) estimated this to be just over 3% of the rate of entry into the DP population, meaning that fueling this trickle of output requires that roughly 30% of all thymocytes enter the DP stage each day. This again illustrates the extent of the filtering of the pre-selection repertoire that appears to be required to produce a functional and self-tolerant population of naive T cells. The thymus gradually involutes and its rate of output declines with age in both mice (23) and in humans (24), indicating that the bulk of the peripheral T cell repertoire is probably generated early in life.

### The majority of thymocyte division likely occurs pre-selection

Labeled nucleotide uptake assays have revealed that substantial proliferation of thymocytes occurs before selection on self-pMHC ligands begins, stopping at or around the time of TCR rearrangement at the late DN/early DP stage (6, 25, 26). However, it is proliferation following TCR rearrangement that is most relevant for understanding how repertoire diversity is generated. Division during selection means a smaller proportion of TCR clonotypes may pass selection than measures of percentage survival suggest (27). The extent of division early in selection is unclear – estimates of the proportion of newly generated DP cells that are dividing have ranged from 11 to 68% (6, 25, 28), and CFSE labeling in *in vitro* thymic organ cultures showed up to 5 divisions from DP onward (29). However, the DP population comprises cells pre- and post-TCR rearrangement, and there appears to be very little proliferation within the more mature DP population (6, 11, 15). There is a low level of proliferation during or just before the SP stage (11, 13, 25, 30), with CD8 SP more prone to division than CD4 SP (10).

Perhaps the most reliable experimental measure of average levels of proliferation during selection uses T cell receptor excision circles (TRECs). TRECs are circular DNA fragments that are stable remnants of the recombination events that generate the TCR and are shared randomly between daughter cells on division. The mean TREC content per cell is a rough measure of the mean number of divisions that have taken place since the TCR was generated. One caveat is that TREC studies are used most commonly in humans and much of what we discuss here derive from studies in mice. Another is that standard TREC measurements contain no information about the variance of the division number, and may gloss over even quite extreme heterogeneity in division patterns. Nevertheless a study of human infants observed 1–2 divisions on average between TCR rearrangement at the CD3^low^ CD4^+^CD8^+^ stage and mature CD4 or CD8 SP; once shortly after TCR rearrangement, and another at the CD8 (but not CD4) SP stage (31). The high TREC content they observed at the early DP stage may reflect multiple rearrangements taking place in order to generate a functional TCRα-chain. In line with these results, the TREC content of naive CD31^+^CD4^+^ recent thymic emigrants in human infants is ~0.1–0.9/cell (32), suggesting that up to three divisions take place on average between TCR rearrangement and export to the periphery, although this may include some post-thymic proliferation and so is an upper limit on the extent of intra-thymic division.

### Turnover rates and transit times

Experimental estimates of the times taken to transit different developmental stages (immature DP → mature DP → SP → Export) are variable, particularly within the SP population (6, 10, 12, 25). Possible reasons for these discrepancies include different labeling protocols, different gating strategies defining thymic subpopulations, heterogeneity of cell populations, and differences in the kinetics of MHC class I-restricted and class II-restricted lineages. It has also been unclear whether selection is a “conveyor belt,” first-in first-out, or has a more stochastic “lucky dip” nature (25). From a modeling perspective these are two points on a continuum. If an experimentally identifiable developmental stage comprises several shorter, sequential differentiation steps, the variance in the transit time through that stage is lowered with respect to a single-step model of transit. The more obligate steps, the more conveyor-belt-like the system appears.

There is general agreement that the transition from non-dividing mature DP to SP takes on average 3–4 days (6, 12, 15, 28), although it has been argued that it takes significantly longer to reach CD8 SP than CD4 SP (33). This transition is dependent on TCR signaling (15, 34). Observing a well-defined delay in the appearance of labeled SP cells, Egerton et al. (6) argued for a first-in-first-out kinetic in the DP population. This suggests DP cells must transit through a number of obligate steps. Subsequent experimental and modeling studies have addressed this, and are discussed below. The same study estimated a mean SP residence time of ~12 days, comparable to other estimates of the medullary residence time (6, 28). McCaughtry et al. (10) argued that this is an overestimate of the time mature conventional SP T cells take to develop, because the SP population is heterogeneous, also containing Treg, NKT, and *γδ* T cells, which turn over more slowly. They estimated SP CD4/CD8 residence times to be 4.4/4.6 days. Saini et al. (33) arrived at similar estimates. As for DP cells, there are may be several developmental stages within the SP population and so it seems unlikely that SP residence times are exponentially distributed.

Stritesky et al. (12) estimated the total rate (cells per unit time) at which cells are negatively selected to be almost six times greater than the rate of positive selection, and found that both processes occur predominantly at the DP stage. Converting these figures into the relative efficiencies of positive and negative selection requires knowledge of how long cells spend in each selecting phase. If indeed positive selection is the more stringent, their result indicates that negative selection must take place over a relatively short timescale within the DP compartment. This is supported by a recent study observing negative selection of DP thymocytes taking place over ~12 h (35).

Interpreting data on transit or residence times can be problematic when both death and differentiation are taking place, as they clearly are at the DP stage(s) of development. If death and differentiation are modeled as independent processes, then at equilibrium transit rates through a compartment are not necessarily the same as turnover rates. If cells are maturing at rate *μ* and dying at rate *δ*, the population turns over at rate *μ* + *δ* and the expected time a cell spends in that compartment is 1/(*μ* + *δ*). However, the mean time that successfully differentiating cells spend in each compartment is shorter because it is conditioned on survival, and is *μ*/(*μ* + *δ*)^2^ (if cells are capable of maturing but are simultaneously at risk of dying, those that successfully mature tend to do so early). This difference can be quite substantial, as we see below.

### Kinetic models of thymic development

Data from these experimental studies and others have invited the use of population dynamic models to infer the kinetics of development. In the first studies to model thymic development, Mehr and collaborators utilized ordinary differential equation (ODE) models of the flow from DN → early DP → late DP → CD4/CD8 SP (36, 37). They utilized measures of steady-state population sizes and parameters either inferred from data or explored systematically to ask questions about the underlying dynamics. Mehr et al. (36) argued that positive selection likely involves triggering of proliferation as well as rescue from death, and while they were unable to use the steady-state data to make strong statements about the timing of positive versus negative selection, they inferred that most death at the DP stage is due to failure to positively select, consistent with many experimental and subsequent modeling studies.

There is evidence from fetal thymic organ cultures that populations of mature CD4^+^ T cells resident in the thymus may enrich for the CD4 lineage while reducing thymic output. Mehr et al. (37) used a similar model with these data to propose that the mature resident cells increase survival of developing single-positive CD4 T cells while reducing proliferation or increasing the rate of differentiation of DP cells. They suggest that mature CD4 T cells exert their influence by restricting the number of available pMHC ligands in the thymus, which could simultaneously reduce proliferation of DP cells (lowering thymic output) and decrease the stringency of negative selection (increasing the efficiency of maturation into the mature SP state). Again, these conclusions were reached using data from the thymus at steady-state.

Mehr and collaborators also studied the seeding of the cortical stroma with bone marrow-derived progenitor cells using a combination of modeling and experiment. They showed how migration between niche sites explained the competitive advantage of younger progenitors over older (38, 39), and that reconstitution of the progenitor population following irradiation is limited by damage to stromal niches and incumbent, surviving cells (40).

Thomas-Vaslin et al. (13) studied naive T cell homeostasis from the thymus through to the periphery. They induced systemic depletion of T cells for 7 days through expression of a suicide gene in dividing cells, and followed the kinetics of reconstitution. To interpret these data they developed a multi-compartment ODE model of T cell development, with a finer-grained treatment of transit through the DN, DP, and SP stages. In their model extensive proliferation occurs through the DN to early DP, with the latter population dividing 5 times. Their best-fitting model assumes all cell death (positive and negative selection) takes place at the late DP stage. They estimated 5% of total thymocytes (DN, DP, and SP) or ~3 × 10^6^ are exported as naive SP cells per day, and that 93% of DP thymocytes are lost, in line with existing estimates, and again suggesting that the bulk of negative selection occurs at DP. The mean times spent overall in the early DP (dividing), late DP (selecting), and SP compartments were estimated to be 1.2, 2.7, and 5.8 days respectively.

Sinclair et al. (11) used a different experimental system, with controllable TCR signaling that allowed arrest and release of cells at the early DP stage, and used a multi-compartment ODE model to quantify transit dynamics and selection efficiencies. Rather than simply early or late, they broke the DP stage into a branched developmental progression defined by the expression levels of CD5 and the TCR (33). In their schema, DP1 thymocytes are pre-selection; progression to DP2 requires a positively-selecting TCR signal; DP2 thymocytes consist of class I- and class II-restricted thymocytes in the first 12–48 h of development; and DP3 thymocytes are predominantly MHC class I-restricted cells that can select into CD8SP only. Thus cells destined for CD4SP transit DP1-DP2 only, and CD8SP transit through DP1, DP2, and DP3.

Sinclair et al. (11) estimated that ~75% in DP1 fail to progress to DP2, reflecting failure to positively select and dying of neglect. Overall, 5% of DP cells become CD4SP and ~2% become CD8SP, and so ~94% of DP cells are lost. They also saw relatively low levels of cell death in the SP compartment. These results suggest again that the bulk of negative selection occurs before cells transition to SP. They saw very little proliferation in their system, using a variety of methods, and so did not model cell division. Mean residence times in DP1 and DP2 were 3.5 and 1.4 days, respectively, with the smaller CD8 lineage spending an additional 7 days in DP3. They estimated 23% of all thymocytes at DP and SP enter the DP compartment per day. These selection efficiencies and the net flux agree with other estimates. Accounting for the selection bias on maturing cells, the model predicts that successful thymocytes spend on average 1.3 days in DP1 + DP2, 4.5 days in DP3. SP4 and SP8 residence times were 5 and 3.7 days, respectively, with very little cell death occurring. Their analysis therefore suggests that CD4SP/CD8SP cells take ~6.3/9.5 days from entry into DP1 to export.

### Migration within the thymus and the timing of positive and negative selection

From the perspective of modelers attempting to connect models of thymocyte dynamics to data, it is important to understand when and where the different phases of development and selection occur. Selection begins in the thymic cortex, where the majority of thymocytes perform undirected random walks (41) encountering pMHC on cortical thymic epithelial cells. Sensitivity to medullary chemokine receptor signals begins to increase immediately following receipt of a positive selection signal and positively-selected cortical thymocytes eventually display rapid, directed motion toward the medulla (41), where they encounter pMHC on medullary thymic epithelial cells and dendritic cells. Negative selection takes place in the medulla (35, 42–44) but also late in migration through the cortex (45) and possibly even throughout development (46). The mapping between these migratory and selecting processes to developmental stages is not clearly defined. Cells undergoing negative selection in the medulla include DP populations (35), indicating that maturation from DP to SP does not coincide precisely with the cortical–medullary transition but further supporting the conclusion that the extensive cell loss at the DP stage comes from failure of both positive and negative selection. Further, antigen-presenting cells in the cortex and medulla appear to differ in their ability to provide positive or negative selection signals, either through differences in pMHC expression or diversity, or levels of co-stimulation (47–51). It seems therefore that negative selection at the DP stage takes place in at least two distinct spatial and TCR-stimulatory environments.

### Models of selection within the cortex and medulla

Motivated by this, Faro et al. (52) took a different perspective; rather than partitioning selecting thymocytes into developmental stages, they used a probabilistic model to describe selection within the cortex and the medulla. They aimed to quantify the number of selecting events, the number of selecting APC encounters and pMHC engagements, and the efficiencies of positive and negative selection in each region. Using the experimental estimates of overall selection efficiencies, and one experimental estimate of the efficiency of negative selection in the medulla, they inferred that most thymocyte death occurs by failure to positive select in the cortex, and cells are ~10 times more likely to be deleted (negatively selected) in the medulla than in the cortex. With these efficiencies, through a parameter search, they were able to infer the number of ligands each thymocyte selects on in each spatial compartment. They came to the striking conclusion that for each cortical thymocyte selection takes places on <60 pMHC ligand interactions, likely in order to achieve in their model the required high level of failure to positively select. However, this needs to be reconciled with the ~3-day mean lifetime of cells at DP1, which suggests cells have far more opportunities to positively select, either through repeated encounters with APC or through repeated rearrangements of the TCRα chain [see Ref. (53) and refs therein], before dying of neglect.

### Identifying the source of the CD4:CD8 lineage bias in thymus

CD4 SP outnumber CD8 SP by roughly 4:1 in the thymi of many species. Using time courses of development in control mice and those lacking MHC class I or class II, Sinclair et al. (11) estimated the CD4 and CD8 lineage-specific selection efficiencies. In control animals, the highest death rate was at the positively-selected DP2 stage, and was substantially greater for MHC class I-restricted cells. MHC class I- and class II-restricted cells are indistinguishable at DP1 and DP2, but they were able to back-calculate the rates of production of precursors of the two lineages after TCR rearrangement, and found they were comparable. This suggests that the CD4:CD8 asymmetry in the thymus derives in large part from more stringent selection acting on MHC class I-restricted cells and not from any significant asymmetry in the predisposition of randomly generated TCR to recognize MHC class I or class II. Theirs is a model of CD4/CD8 lineage commitment in which the ability of a DP thymocyte to recognize MHC class I or class II dictates whether it will progress to the CD8 or CD4 lineages, respectively (8, 54). This is contrast to a less efficient, selective process in which a thymocyte’s decision to downregulate either CD4 or CD8 expression is stochastic and decoupled from MHC preference, such that potentially viable TCR may fail positive selection [see, for example Ref. (55, 56); and Ref. (57) for a discussion of a hybrid mechanism]. Mehr et al. (36) proposed a purely instructive model of selection, in which pre-selection thymocytes are in principle able to recognize both MHC class I or II, and concluded that the most likely explanation of the CD4 bias is a difference in the *per capita* rates of maturation from DP into the two lineages, rather than differences in death rates.

The majority of models discussed here assume that thymocytes undergo screening independently. Mehr et al. (36, 37) implicitly allowed for competition with density-dependent proliferation rates at each developmental stage. However, there is some evidence that the probabilities of maturation can be impacted by competition between thymocytes, both globally and in lineage-specific ways. The efficiency of selection of transgenic TCRs varies with their abundance and with the availability of cognate pMHC (15, 58–60), and the selection of polyclonal MHC class I-restricted thymocytes is more efficient in the absence of MHC class II and vice versa (11). These observations suggest that selection efficiencies may be limited by competition both within and between lineages for access to pMHC or other resources needed for selection, and so may impact on the CD4:CD8 ratio emerging from the thymus. Two studies have used explicitly spatial, agent-based models of thymocyte migration and development to investigate this issue. Souza-e Silva et al. (61) modeled the movement of DN, DP, and CD4 SP and CD8 SP populations and their interactions with thymic epithelial cells (TEC) and chemokine gradients, using a 2D model. The structure of the epithelial networks was derived from histological samples from both mice and infant humans. Parameters were chosen to give agreement with published data regarding the repopulation of the thymus after sublethal irradiation, although a sensitivity analysis was not performed. In their model the CD4:CD8 ratio emerges as a result of competition for access to TEC and stochastic variation in the duration of signaling, which has been associated with CD4/CD8 lineage commitment (62). Their simulations also reproduce an observed variation in the CD4:CD8 ratio as irradiated thymi reconstitute and, in their model, the degree of competition increases. Efroni et al. (63) also took an agent-based approach and concluded that MHC class I and class II ligands on TECs are limiting. If continued access to pMHC stimulation is required for survival, and class I restricted cells stay conjugated to MHC for longer than MHC class II-restricted cells, exclusion of competitors leads to a higher death rate of cells developing into the CD8 lineage and a skewing of the CD4:CD8 ratio. Such a competitive model is an experimentally testable explanation of the differential death rates observed by Sinclair et al. (11).

## Characteristics of the TCR Repertoire

Various summary statistics can be used to describe T cell populations pre- or post-selection. The *diversity* (or the *repertoire*) usually denotes the total number of distinct TCR sequences or clonotypes. The *cross-reactivity* measures a TCR’s capacity for discrimination, and is quoted as either the average number or the proportion of different pMHC that one TCR responds to above some defined functional threshold. *Specificity* is inversely related to cross-reactivity. A mirror quantity is the *precursor frequency*, also referred to as the response frequency – the average proportion of all TCR capable of recognizing one pMHC. Further, selection operates in the context of an individual’s own MHC alleles. *MHC restriction* measures the degree to which a given TCR is limited to recognizing peptides presented by one or more self-MHC; and *alloreactivity* is the proportion of TCR that respond to a foreign MHC, which is relevant for transplantation of tissues from one individual to another. In the sections that follow we describe how theoretical models have been used to understand how these quantities are linked and constrained by thymic selection.

### TCR cross-reactivity

A diverse TCR repertoire seems to be a requirement for coverage of pMHC shape space. However, the number of theoretically possible pMHC complexes appears to be far greater than any individual’s capacity for unique TCR clonotypes (64–66); a simple calculation for just one MHC class I variant, assuming it presents 2% of all possible 9-residue peptides, yields $20^{9}\times 0.02≃10^{10}$ possible pMHC, compared with the roughly 5 × 10^7^ naive CD8 T cells in a mouse. To minimize the probability that any given foreign pMHC will escape detection by the immune system, some degree of TCR cross-reactivity therefore seems beneficial. Mason (64) used a variety of methods and data sources to estimate that one MHC class I-restricted T cell responds to between 10^6^ and 10^7^ nonamer peptides, or one in 10^3^ to 10^4^ pMHC using the theoretical estimate of the potential pMHC diversity; and Ishizuka et al. (65) used peptide libraries to estimate more directly that one CD8 T cell clone responds to roughly 1 in 3 × 10^4^ peptide–MHC class I ligands. On the other hand, the average degree of cross-reactivity seems necessarily constrained from above, to avoid excessive deletion of the repertoire and to preserve specificity for self/non-self discrimination. It therefore seems plausible that evolutionary pressures might have optimized this trade-off and determined the degree to which TCR can respond to multiple pMHC.

### Optimal levels of TCR cross-reactivity – probabilistic arguments

Several variants of essentially the same argument predict that the diversity of self-peptides involved in selection is the strongest influence on the optimum level of TCR cross-reactivity (64, 67–70). One version of the argument is as follows. The proportion of the positively-selected T cell repertoire *R*_0_ that avoids deletion, *f*, decreases with both the number of self antigens *N*_*s*_ and the cross-reactivity *r*, $f={\left(1-r\right)}^{N_{s}}$ which is approximately $\exp\left(-rN_{s}\right)$ for $\mathrm{r ≲}1∕N_{s}$. A pathogen escapes immune recognition if all *f R*_0_ surviving unique clonotypes fail to recognize (cross-react with) all *x* epitopes it generates, with probability

(1) $$P_{E}={\left(1-r\right)}^{fR_{0}x}≃\exp\left(-rfR_{0}x\right)$$

where again the approximation holds if *r* ≲ 1/(*f R*_0_*x*). This ignores MHC restriction, but including this refinement yields similar conclusions (67). Using the expression for *f*,

(2) $$R_{0}≃-\log\left(P_{E}\right)\frac{\exp\left(rN_{s}\right)}{rx}.$$

This equation connects the repertoire before negative selection *R*_0_, the probability of immune escape *P*_*E*_ and the pre-selection cross-reactivity *r*. *R*_0_ is relatively insensitive to *P*_*E*_ but very sensitive to the diversity of self, *N*_*s*_. In this model, then, the strongest determinant of the size of the pre-selection repertoire is the diversity of self antigens, *N*_*s*_, and not the requirement for minimizing the probability that a pathogen escapes detection (67).

The three-way relation expressed by equation (2) can then be used to estimate the optimal cross-reactivity under different evolutionary constraints. Suppose the potential repertoire size *R*_0_ is relatively conserved and evolution has selected for the smallest *P*_*E*_ by tuning TCR cross-reactivity; in this case, the optimal cross-reactivity is simply the inverse of the number of distinct self-pMHC involved in selection, *r* = 1/*N*_*s*_. The same value of *r* arises if evolution is assumed to minimize the required repertoire size *R*_0_, whatever the value of *P*_*E*_ (67). Thus the more diverse the self-peptides involved in thymic selection, the more specific (less cross-reactive) the TCR needs to be. The same result can be derived in a very general way using extreme-value theory (70), requiring only the assumption that the negative selection threshold in the thymus is equal to the activation threshold in the periphery.

The induction of tolerance in the thymus is likely incomplete and there may be mature lymphocytes that are able to recognize self-peptides not involved in thymic selection. Borghans and De Boer (71) argued that to minimize the probability of these cells mounting a cross-reactive autoimmune response to this “ignored self” while responding to a pathogen demands higher levels of specificity than predicted by the simplest models. In this model, optimal cross-reactivity is then modulated by the potential diversity of the repertoire; the greater the number of possible T cell clonotypes, the lower cross-reactivity is required.

Percus et al. (72) took a different approach to studying optimal cross-reactivity, prompted by the observation that the sizes of the binding sites of the TCR and the B cell receptor (antibodies) are similar, at roughly 15 amino acids. They concluded that this size is large enough to provide discriminatory power but small enough that there is sufficient cross-reactivity for coverage of foreign antigen shape space. Interestingly this result does not arise from the demand for self–non-self discrimination, but rather from the constraint of the observation that the B and T cell repertoires comprise ~10^7^ different receptors. However, this diversity itself may be derived from the self-tolerance arguments described above (64, 67–69). It has since been established that substantially fewer peptide residues are involved in TCR recognition. Burroughs et al. (73) analyzed the proteomes of humans and several microorganisms and showed that even the seven exposed (non-anchor) residues of the nine-mer peptides bound to one MHC class I allele may promote self/non-self discrimination, with <0.5% overlap in these sequences between humans and different microorganisms.

### Convergent estimates of levels of negative selection

Several of these studies concluded that at the optimal level of cross-reactivity the probability of negative selection is roughly 63%, making various assumptions regarding the magnitude of parameters and maximizing the probability that the post-selection repertoire mounts a response to a foreign pMHC. However, the probability of negative selection can be derived without any assumptions regarding parameter values. From above, the fraction of the positively-selected repertoire with cross-reactivity *r* that survives deletion on *N*_*s*_ self-peptides is $f={\left(1-r\right)}^{N_{s}}$. The probability that the post-selection repertoire *R* = *fR*_0_ fails to recognize one given foreign pMHC is given by equation (1) with *x* = 1,

(3) $$P_{E}={\left(1-r\right)}^{fR_{0}}={\left(1-r\right)}^{R_{0}{\left(1-r\right)}^{N_{s}}}.$$

This is minimized with respect to *r* at *r* = 1 − exp(− 1/*N_s_*), exactly (the optimal cross-reactivity $r≃1∕N_{s}$ then obtains if $N_{s}\gg 1$). So if evolution acts on cross-reactivity to minimize the probability of foreign pMHC escaping detection, the fraction of the positively-selected repertoire that survives negative selection is then simply $f={\left(1-r\right)}^{N_{s}}=\exp\left(-1\right)≃0.37$, or ≃63% of positively-selected thymocytes are deleted.

Mason (64) arrived at the same result assuming heuristically that the quantity to be maximized is the “reactivity” of the repertoire, proportional to the number of peptides each T cell can recognize multiplied by the proportion surviving negative selection;

$$\begin{array}{llll} & \mathrm{Reactivity}∼\text{Cross-reactivity}\; & & \;\\ & \;\times P\left(\mathrm{survive negative selection}\right)∼r\times {\left(1-r\right)}^{N_{s}}.\; & & \; \end{array}$$

Maximizing this reactivity is equivalent to minimizing the probability of escape in equation (3) when *r* is assumed to be small. There, using the Taylor expansion gives $P_{E}≃1-rR_{0}{\left(1-r\right)}^{N_{s}}$, and so the probability of responding (1 − *P*_*E*_) is ~$rR_{0}{\left(1-r\right)}^{N_{s}}$, or Mason’s reactivity. Since *r* is small, the probability of negative selection is ${\left(1-r\right)}^{N_{s}}≃\exp\left(-rN_{s}\right)$ and so the reactivity is proportional to *r* exp(*−* *rN_s_*), which is maximal with respect to *r* when argument of the exponential is −1. Thus again f≃0.37 and the optimal cross-reactivity $r≃1∕N_{s}$.

An essentially identical argument applies to negative selection of B cells (67, 69). This estimate of *f*  is remarkably consistent with estimates of levels of negative selection in the thymus from several experimental and population dynamic modeling studies (11, 17, 19–22).

### Alternative treatments of cross-reactivity

These models assume a universal cross-reactivity parameter *r*, but T cells may have the capacity to modulate their activation thresholds in response to their signaling environment (74, 75). Motivated by this, Scherer et al. (76) developed a model in which T cells tune their activation thresholds (and thus their cross-reactivity) to the level of their strongest interaction with self-pMHC during selection. If combined with a deletion mechanism that removes cells with activation thresholds so high as to be judged functionally inert, this model appears to be a more efficient mechanism of thymic selection than the standard clonal deletion model. Scherer et al. showed that the tuning model increases the probability of mounting an immune response to a given pathogen epitope, given a pre-selection repertoire size *R*_0_, and the number of self-pMHC ligands involved in selection, *N_s_*. The improvement offered by the tuning model is most striking for small pre-selection repertoires, $R_{0}\ll N_{s}$, but disappears for $R_{0}\gg N_{s}$. The latter inequality likely holds for mice and humans; the potential number of unique TCR sequences exceeds the estimated 10^3^–10^5^ self-peptides able to be presented by a given MHC allele (73, 77, 78). Further, equation (1) predicts that at the optimal cross-reactivity *r* = 1/*N_s_*, the probability of one epitope (*x* = 1) escaping recognition is $P_{E}=\exp\left(-R_{0}∕eN_{s}\right)$ where *e* is the base of the natural logarithm. For *P*_*E*_ < 0.05, expected in humans and mice, requires $R_{0}≳10N_{s}$. Despite this, Scherer et al. (76) argue that the tuning model is a more parsimonious mechanism of self-tolerance in the thymus than the standard model of deletion based on evolutionarily optimized cross-reactivity.

Finally, many of these arguments assumed thymic selection alone optimizes cross-reactivity, but the requirement for memory T cells to discriminate between different pathogens may impose a further constraint of its own (79, 80).

### Exploring cross-reactivity with sequence-based models of thymic selection

A series of related papers by Detours, Perelson, and Mehr (27, 81–83) used a model of TCR–pMHC interactions to understand at a more mechanistic level how cross-reactivity, alloreactivity, and MHC restriction emerge in the post-selection repertoire. Here we focus on their treatment of TCR cross-reactivity, and return to alloreactivity and MHC restriction in the next section. Their starting point was an established model of protein binding (81, 84). They described the interaction between the variable region of the TCR and its pMHC ligand with strings of digits, and binding strengths between each digit pair were determined by the degree of complementarity between their binary representations (81). MHC and peptide contributed additively to the affinity of the interaction, the quantity assumed to drive selection. Given the number of digits ascribed to the polymorphic MHC residues in contact with the TCR, and the number of digits representing the peptide, selection could be performed on a randomly generated TCR repertoire using randomly generated peptide–MHC complexes. Affinity thresholds were then adjusted to give stringencies of positive and negative selection similar to those observed experimentally.

To circumvent the computational costs of selection using realistic numbers of peptides and unique pre-selection TCRs, they derived expressions for the mean-field predictions of the model for given parameter sets. This has the advantage of yielding population-level statements, which average over all possible TCR, MHC, and peptide sequences.

Detours and Perelson (82) estimated the precursor frequency, the proportion of naive T cells able to respond to a particular foreign pMHC. Experimental estimates of this quantity lie in the range 10^−6^–10^−4^ (85–89). They term this the response frequency, *R*, and found it to be strongly and inversely related to the number of selecting self-pMHC ligands. Since precursor frequency is positively correlated with cross-reactivity (64), this result is in keeping with the theoretical studies discussed above (64, 67–70). It is also consistent with observations that repertoires selected on a restricted range of peptides exhibit higher cross-reactivity than normal (90–92). For *R* to lie in the observed range constrains the number of distinct peptides each MHC can present to be of the order 10^3^–10^5^, in line with estimates for murine MHC class I (77), MHC class II (78), and human MHC class I (73).

To explore the effect of thymic selection on specificity in more detail, Chao et al. (93) revisited the complementary digit-string model. Again peptide and MHC were assumed to contribute additively to an antigenic distance from the TCR, which was inversely related to affinity or the strength of a selecting signal. They confirmed that negative selection reduced the coverage of peptide space, defined as the proportion of peptides that are recognized on the selecting MHC. This was equivalent to a reduction in the cross-reactivity of the repertoire; it reduced the mean antigenic distance to foreign pMHC complexes.

Chao et al. (93) then used the model to address the question of why the number of pMHC that one T cell is able to respond to varies widely across TCR (94). Their simulations suggested that the degree of cross-reactivity to a foreign peptide was inversely related to the peptide’s similarity to self, which can be understood with the following argument. In their model, in the pre-selection repertoire a TCR’s affinity for the MHC and peptide portions of its ligand are uncorrelated. Selection introduces an inverse correlation between a TCR’s affinity for its selecting MHC and its strongest affinity for self-peptide; to select, a TCR’s strongest interaction with self must lie between the positive and negative selecting thresholds. (The narrower the range of affinities defining the selecting region, the stronger this correlation will be.) Selected T cells with high affinity for MHC then have a relatively low affinity for the self-peptide component and require only weak binding to foreign peptide to be activated (activation in their model is defined to be an interaction above the negative selection threshold). These cells are therefore cross-reactive to foreign peptides. Conversely, selected TCR that bind relatively weakly to MHC have higher affinity to self and require strong binding to foreign peptide for activation, and therefore have more specificity for foreign antigen. Thus it emerges from their model that a TCR’s specificity to foreign peptide is positively correlated to its affinity for self-peptide; or equivalently, a TCR’s cross-reactivity is positively correlated with its affinity for MHC.

The effect of negative selection on cross-reactivity can be understood with a similar argument. A TCR with high affinity for MHC will survive negative selection only if it has low affinity to all self-peptides, which is unlikely. Negative selection therefore enriches for cells with lower affinity for MHC, which from the argument above tend to be less cross-reactive. This reduction in coverage means specificity to foreign peptide must be increased.

Kosmrlj et al. (95) used a more physical, mechanistic approach to understanding how negative selection increases specificity, with the aim of characterizing the properties of the amino acid sequences of specific and cross-reactive TCR. Using the Miyazawa–Jernigan matrix (96) to quantify the interaction energies of pairs of amino acids, they extended the digit-string model to calculate the binding affinities between the peptide and the CDR3 region of the TCR, with a constant contribution from the MHC. (The variable peptide element of the pMHC ligand can be assumed to include the polymorphic MHC residues; thus their model may allow for MHC restriction, although this was not discussed.) Košmrlj et al. (97) presents an analytical treatment of the model.

They observed that TCRs selected against multiple peptides on the same MHC had peptide contact residues enriched in weakly interacting amino acids. In their model this arises by a sort of buffering mechanism – such sequences are able to withstand multiple substitutions in the peptide sequence to which they bind most strongly, and so are more resistant to negative selection than those TCR with strongly binding residues. For these TCR to survive selection requires that the invariant MHC contribution to the binding energy is of moderate strength – contributing sufficiently to favor positive selection but well below the negative selection threshold.

Kosmrlj et al. (95) argue that it is this enrichment for weakly binding TCR driven by negative selection that underlies antigen specificity. Antigen recognition is assumed to occur when a TCR signal exceeds the negative selection threshold made up by several interactions. This requires the peptide to contain several amino acids capable of binding the most strongly to the generally weakly binding TCR contact residues. Each contributes significantly to the total binding energy, and so any mutation to the peptide sequence has a high probability of abrogating recognition. Thus there is a restricted peptide signature or “barcode” required to trigger the TCR. In their model, TCR selected against a single pMHC were enriched slightly for strongly interacting amino acids. For these TCR, they argue, fewer amino acids contribute on average to the binding energy, triggering is more robust to mutations in the peptide sequence, and so the TCR is more cross-reactive. Thus again the argument emerges that the cross-reactivity is inversely related to the diversity of self driving selection. Kosmrlj et al. (98) employed this idea to put forward an explanation of why the population of elite-controllers of HIV infection is enriched for the HLA-B57 allele. Using a predictive peptide binding algorithm they argued that HLA-B*5701 binds a lower diversity of self-peptides than average. Cytotoxic T cells restricted to this allele are then expected to be more cross-reactive than average and so are more resistant to virus mutations that might otherwise escape CTL control.

Chao et al. (93) and Kosmrlj et al. (95) took different approaches to the problem of how negative selection increases specificity. They came to the common conclusion that the most specific TCR are those with low to intermediate affinity to MHC – high enough to have a reasonable probability of passing positive selection, but low enough to avoid negative selection by allowing headroom for the additional contribution from the peptide component. The greater this headroom, the smaller the proportion of peptides that can trigger activation and so the greater the specificity.

### The emergence of specificity in avidity-based models of selection

Van den Berg et al. (99) developed a statistical framework to study the question of how specificity and self-tolerance can derive from a pre-selection repertoire of relatively promiscuous TCR. In their formalism, T cell activation is avidity-based and related to the rate of TCR triggering. Their starting point is that TCRs are degenerate and low affinity, binding weakly to many pMHC. TCR perceive an average signal derived from endogenous self-pMHC, and are triggered only by pMHC with sufficiently high prevalence and affinity to be visible above this background. The authors introduce the concept of an antigen presentation profile (APP), characterizing the abundances of different pMHC on antigen-presenting cells (APC). Positively-selected cells are selected against a given number of APC each with distinct APPs. In their framework, negative selection acts only on ubiquitous peptides presented on all APCs, and decisions are made on the basis of the entire APP of one APC. TCR that are triggered by this constitutive self-background are deleted. This filtering acts to sharpen the boundary between triggering rates, which give low and high activation probabilities, and so specificity can emerge even from a highly degenerate TCR. Interestingly they predict that negative selection does not have to be particularly stringent to generate an acceptably self-tolerant repertoire. Nevertheless in this model the selected repertoire may still be reactive to self-peptides expressed heterogeneously in the thymus, and in particular to peptides expressed at high levels only on certain cell types. Van den Berg and Rand (100) review avidity-based models of ligand discrimination.

### Alloreactivity and MHC restriction

A high proportion (1–24%) of peripheral T cells are reactive to peptides presented on a foreign MHC allele (101–103), reflected clinically by acute T cell mediated rejection of grafts from MHC-mismatched donors. These promiscuous “allogenic” responses contrast with the low precursor frequency (10^−6^–10^−4^) in normal immune responses to peptides presented by an individual’s own MHC. Allogenic responses are also apparently counter to the notion of MHC restriction. Reconciling these results may tell us great deal about the relative contributions of peptide and MHC binding motifs to the TCR signals driving selection, and how this breakdown influences the coverage and cross-reactivity of the T cell repertoire.

Detours and Perelson (82) used their digit-string model of TCR–pMHC interactions, described above, to show how the probabilities of responsiveness to self and foreign MHC emerge. Mean alloreactivities of 1–2% arose naturally, at the lower end of the range of experimental estimates, and they showed that the alloreactivities of the pre- and post-selection repertoires are similar, as observed experimentally (17, 22). In essence, the modeling supports the hypothesis that the greater degree of alloreactivity than response frequency arises simply because many more pMHC ligands can be generated from one MHC than can be generated from one peptide (104). In other words, each TCR is triggered by ligands in a subset of pMHC shape space; one particular MHC along with its associated diversity of peptides will cover a far greater region of shape space than covered by one peptide and all the self-MHC alleles capable of presenting it; a given MHC will then stimulate far more of the T cell repertoire than will a given peptide.

They found that alloreactivity correlates with the extent of negative selection and inversely to the degree of MHC restriction. It can be seen intuitively how this emerges from their model. If negative selection is weak, positive selection must be correspondingly stringent in order to yield the selection efficiencies observed experimentally (3–5%). Stringent positive selection imposes an imprint of self-MHC on the repertoire – only those TCRs that bind strongly to self-MHC residues survive. The strength of binding to a randomly generated MHC not involved in selection (i.e., a foreign MHC) is then on average lower to that of self-MHC in the post-selection repertoire. This difference increases, and thus alloreactivity decreases, as the required strength of binding to self-MHC increases.

This trade-off between alloreactivity and restriction might be expected as they appear to be in conflict. However, experimental estimates of these two quantities are variable. The conclusions described above were derived analytically from a model capturing the mean-field behavior of the digit-string selection process, but did not deal with the variance in these measures of the repertoire outputs across specific simulations or experimental systems. The final study of the series (83) took a simulation-based approach, explicitly performing repertoire selection on random TCR and pMHC populations. This confirmed the inverse correlation between alloreactivity and MHC restriction and yielded sufficient variability to account for restriction ranging from absolute to partial in different settings.

Overall the digit-string model explored by Detours and colleagues yields remarkable agreement with many observations. Their model of TCR–pMHC binding is highly abstracted, but appears to be a powerful one. In part this might be because the relevant quantities for selection in their model are the minimum and maximum binding affinities that each TCR experiences during exposure to large samples of randomly generated pMHC strings. These two quantities will be drawn from extreme-value distributions, which should be insensitive to the distribution of binding strengths of randomly chosen TCR–pMHC pairs (70, 105). The additivity of the MHC and peptide contributions to the fate-determining signal is likely the most questionable assumption, as the authors point out. Fate decisions may be based on the sum of several TCR interactions (which means for example that positive selection may occur though proximal binding of multiple low-affinity ligands) and so an avidity-based model may be more appropriate. Another caveat is that the population-average model assumes that positive selection takes place on at most one MHC allele, which we will also return to.

### Insights into fate determination mechanisms from stochasticity in selection

Regulatory T cells (Treg) are a distinct lineage of CD4SP cells thought to lie at the higher end of the spectrum of acceptable self-reactivity and play a crucial role in the control of autoimmunity and tolerance to innocuous antigens. Many experimental studies of Treg development have shown that cells with the same TCR can develop into conventional and regulatory T cells within the same selecting environment [see, for example, Ref. (58, 106)], illustrating again, as represented in so many models, the stochastic nature of selection. There are at least two possible sources of this stochasticity. In a purely selective model precursors with identical TCR might be predisposed to the conventional or Treg fates through natural variation in expression of factors involved in lineage commitment. In a purely instructive model, cells within a clone are uncommitted, and intra-clonal heterogeneity in fate may derive from variation in the experience of each thymocyte during selection – most likely because each encounters a different random sample of self-peptides.

Bains et al. (107) used a probabilistic, instructive model that reflects this view of fate determination driven entirely by antigenic experience during selection, in conjunction with data from Ref. (58) to infer the number of pMHC binding events involved in fate determination. In that study, the numbers of conventional and Treg cells with a transgenically expressed TCR were measured for varying abundances of that TCR’s agonist peptide on thymic epithelial cells. Conventional cell numbers declined monotonically with agonist abundance, while Treg increased and then decreased. Thus as agonist abundance increased, it appeared that T cells were initially diverted into the Treg lineage, before the risk of deletion through exposure to agonist dominated. Using this information and a simple graphical argument they were able to infer that fate decisions could not be affinity-driven (that is, made on the basis of a single pMHC interaction) unless TCR sensitivity varies during development, for which there is evidence [see Ref. (107) and references therein]. This model also explains apparently paradoxical observations regarding the effect of partial and full TCR agonists on the efficiency of Treg production (108).

### The limits of negative selection

The potentially very large number of unique self-pMHC prompts the question of whether it is possible to tolerize thymocytes to all self-peptides within the timescale of thymic development. Müller and Bonhoeffer (109) studied this problem. Using constraints from the mouse proteome and the efficiencies of peptide production and binding to MHC, they estimated an upper limit of approximately 5 × 10^6^ possible self-pMHC class I complexes. Notably, this diversity of self is several orders of magnitude lower than figures derived from the simple combinatoric arguments (64, 66) and is more closely aligned with an estimate that ~10^5^ different nine-mers derived from the human proteome are expected to bind to one human MHC class I allele (73). The key quantity in Müller and Bonhoeffer’s calculation is the probability *P* that a given self-pMHC is presented by any given APC in sufficient numbers for negative selection to occur. The probability that a thymocyte specific for this (and only this) self-pMHC escapes negative selection is *P*_E_ in their notation – distinct from the probability of immune escape discussed above – and *P*_E_ = (1 − *P*)*^n^*, where *n* is the number of unique APC encountered during selection. In this model, *P*_E_ is extremely sensitive to the number of copies *h* of a given self-pMHC that an APC needs to present in order to cause deletion – varying *h* between 15 and 1500 gives values of *P*_E_ between 10^−11^ and 0.8. Favoring the higher estimates of *h*, Müller and Bonhoeffer (109) concluded that negative selection on the potential diversity of self is likely to be very leaky. Instead, they suggest thymic selection operates on a restricted subset of self-pMHC, a constraint imposed by the number of APCs encountered during selection. This requires that further tolerogenic mechanisms operate in the periphery to prevent autoimmune response to self antigens not encountered in the thymus (53, 70).

To support their argument, Müller and Bonhoeffer (109) reverted to the older model of cross-reactivity and selection to generate another estimate of the number of selecting ligands using the observed efficiency of negative selection. Recall that the probability of thymocyte with cross-reactivity *r* escaping negative selection on *N*_*s*_ unique selecting ligands is $P={\left(1-r\right)}^{N_{s}}≃e^{-rN_{s}}$. Using the estimate of *r* = 2 × 10^−5^ (88), and P≃0.33, they obtain $N_{s}≃10^{5}$ unique selecting self-pMHC, or ~4% of the putative total number of self-pMHC. This estimate is consistent with those of Detours et al. (27). Both studies assume that this cross-reactivity *r* of thymocytes with self-pMHC is equal to the cross-reactivity of mature naive T cells to foreign pMHC. Since negative selection likely acts as a filter to reduce cross-reactivity in the post-selection repertoire (see above), this assumption is moot. But the need to meet the empirical constraint $e^{-rN_{s}}≃0.33$ implies that higher values of *r* would reduce the number of unique selecting ligands *N*_*s*_ even further.

A subsequent exchange (110, 111) discussed the assumption that each TCR negatively selects only on a single self-pMHC ligand. Müller and Bonhoeffer (111) argued that in the Bernoulli trial model of cross-reactivity and selection, a 33% probability of survival implies that another third of all thymocytes were reactive to one self-pMHC only, giving some quantitative support to their original model. The discussion also addressed whether *N*_*s*_ is constrained by the residence time in the thymus or is a result of restricted presentation of self antigens. Müller and Bonhoeffer (111) favored the latter, presuming that evolution has optimized the thymic residence time for the purposes of efficient selection on a subset of self-peptides. More recently it has been argued that incomplete depletion of self-reactive cells in the thymus may be sufficient for robust self/non-self discrimination in the periphery, if interactions facilitating consensus between T cells are required for the initiation or suppression of immune responses (70).

## Optimality of Individual MHC Diversity – Constraints Arising from Thymic Selection

The polymorphism of the MHC is huge, with hundreds of alleles identified at the HLA-A, HLA-B, and HLA-DR loci in humans (MHC is referred to as HLA in humans but hereon the term MHC is generally used, for simplicity). This diversification is thought not to have occurred by genetic drift but by two non-exclusive mechanisms. Heterozygote advantage (112, 113) suggests that individuals expressing more unique MHC alleles gain fitness by being able to present a larger array of pathogen peptides. Overall the evidence for heterozygote advantage in experimental models of infection is equivocal, though, and it has been argued with a quantitative model that this mechanism alone is insufficient to explain the extent of allelic diversity (114). Another theory is that MHC polymorphism is maintained by frequency-dependent selection under pathogen pressure, in which rare alleles confer protection against pathogen subversion of peptide presentation by commonly expressed alleles (115).

Intriguingly, individuals possess only a small proportion of all MHC alleles. Heterozygous humans possess six at the major HLA-A, HLA-B, and HLA-C loci, which code for MHC class I molecules that present peptides to CD8^+^ T cells, and six to eight at the HLA-DP, HLA-DQ, and HLA-DR MHC class II loci, which present to CD4^+^ T cells. A common explanation for this restricted within-individual diversity is that it derives from the need to generate a broad, functional, and self-tolerant TCR repertoire in the thymus without excessive negative selection (116, 117). The qualitative argument is as follows. If *n* is the number of MHC alleles per person, then increasing *n* both increases the diversity of pathogen-derived peptides that can be presented and increases the probability that a thymocyte will be able to obtain positively-selecting signals. On the other hand, higher *n* will also increase the range of self-peptides that can be presented. This will increase the stringency of negative selection, leading to inefficient generation of T cells in the thymus and potential gaps in the repertoire’s coverage of peptide space. The observed number of different MHC molecules per individual may result from a trade-off between these demands.

The nature of MHC restriction needs to be considered carefully in these arguments. If restriction is absolute and each TCR recognizes only one MHC allele, increasing the number of alleles per person simply increases the size and diversity of the T cell repertoire with no cost because selection operates on each MHC-restricted subset of the pre-selection repertoire independently. In this case an upper limit to within-host MHC diversity might derive only from the need for APC to display sufficient numbers of peptides in conjunction with each MHC molecule to reliably mediate selection or immune activation. The trade-off evident in the qualitative argument above arises when MHC restriction is not absolute and thymocytes are capable of being positively and/or negatively selected on more than one allele.

Woelfing et al. (118) provide an excellent review of theoretical approaches to understanding intra-individual MHC diversity, but we outline the key results here. Nowak et al. (119) were the first to assess the qualitative trade-off argument using a mathematical model. In their analysis they defined *h* and *f* to be the proportions of T cells capable of being positively and negatively selected, respectively, by a given MHC allele. If an individual expresses *n* distinct MHC alleles, they argue that the proportion of the T cell repertoire surviving selection is

$$\left(1-{\left(1-h\right)}^{n}\right){\left(1-f\right)}^{n}.$$

The first term represents positive selection; (1 − *h*)*^n^* is the probability that a TCR fails to be selected by any MHC. The second term represents negative selection; (1 − *f*)*^n^* is the probability that a TCR is not negatively selected by any MHC. The proportion of the repertoire surviving is maximized at *n* = (1/*h*)log(1 + *h*/*f*). They argue that *h* ≤ *f*, supported by the experimental and modeling consensus is that positive selection is more stringent than negative selection. This gives *n*~1/*f*. However, using only the assumptions that hn≪1, or that it is rare for a TCR to be positively selected on more than one MHC allele, and that the proportion of all peptides that can bind to a given MHC is ≪1, they calculate that *n* = 2/*f* maximizes the probability of a response to a randomly chosen foreign pMHC.

Borghans et al. (120) pointed out that this model contains an inconsistency, which allows for cells that fail to be positively selected on one MHC to be negatively selected by the same MHC. They denoted *p* and *n* to be the unconditional probabilities that one TCR is positively and negatively selected by a given MHC molecule. Then *n* < *p*, because the number of cells that fail negative selection on one MHC is necessarily smaller than the number that audition for it following positive selection on that same MHC. The proportion of the original repertoire that survives is then

(4) $${\left(1-n\right)}^{M}-{\left(1-p\right)}^{M}.$$

This model effectively lowers the stringency of negative selection expressed in Nowak et al. (119) and so reduces the cost of increasing the number of MHC alleles. They estimated the probabilities *p* and *n* were 0.01 and 0.005 respectively, using the known efficiencies of positive and negative selection in mice with known numbers of MHC alleles. The optimal value of *M* for these parameter values is far larger than observed allele numbers; conversely, asking what values of *p* and *n* correspond to the observed ranges of *M* being optimal leads to unrealistic levels of positive and negative selection. Their analysis therefore questions the trade-off hypothesis as an explanation of limited MHC diversity.

They suggest alternatives. They estimate that existing typical numbers of MHC alleles together with TCR cross-reactivity may be “good enough” for maximizing the probability of responding to a foreign peptide on self-MHC – in this case the selective pressure for increasing MHC alleles is weak or absent. Alternatively, increased numbers of MHC alleles may increase the risk of autoimmunity through cross-reactivity of T cells responding to antigen that have not been fully tolerized to self. Finally, limited numbers of MHC alleles may allow for sufficient densities of pMHC on the surface of antigen-presenting cells to be able to efficiently select and activate MHC-restricted T cells.

MHC restriction is not absolute in the models described above, although it holds approximately for positive selection when the per-allele positive selection probability *p* is small. However, there is evidence to suggest that MHC restriction is not manifest strongly at the positive selection stage. Zerrahn et al. (22) observed that a relatively large proportion of TCR still positively select when a single type of pMHC was expressed in the thymus. In that study, pre-selection TCRs had approximately a 5% chance of responding to a given class II MHC, independently for different alleles, validating one of the assumptions of these simple probabilistic selection models. On similar lines, Huseby et al. (92) found that the positively-selected repertoire contains TCR with a high degree of cross-reactivity across MHC alleles, and suggested that MHC restriction emerges as a result of negative selection. Finally, the high degree of alloreactivity suggests that positive selection is only weakly MHC-restricted, and that failure to positive select reflects a generic inability to bind to MHC.

Motivated by this possibility, Woelfing et al. (118) revisited these probabilistic models. They assumed positive selection is highly degenerate with respect to MHC and that even very weak cross-reactivity with any allele is sufficient. Under this assumption, one of the presumed advantages of high MHC diversity is removed. Maximizing the probability of mounting an immune response, they estimated the optimal MHC diversity to be in a physiological range of 3–25.

Van den Berg and Rand (121) used a very different and sophisticated approach to the same optimality problem using a mechanistic, stochastic model of TCR triggering rather than the probabilistic repertoire-based models described above. Considering negative selection only, they concluded that limited individual MHC diversity is beneficial for self–non-self discrimination. The essence of their mathematical argument is that restricting the “diversity of foreign” is the key to increasing the signal-to-noise ratio for aTCR attempting to discriminate a foreign peptide from the background of self. This is achieved with a combination of limiting the number of MHC alleles each TCR can recognize (MHC restriction) and limiting the number of peptides that can be presented from one protein on one MHC allele (“peptide selectivity”) to be roughly one. However, the need to ensure that every foreign protein is represented requires multiple MHC alleles, placing a theoretical lower bound on their number. An upper bound comes from the requirement that the density of relevant pMHC ligands must not fall too low on the surface of an APC, similar to the suggestion in Borghans et al. (120) – if a given pMHC is diluted by too many MHC, the relevant TCR will experience fluctuations in signaling that may reduce its discriminatory power. They conclude that of the order 10 MHC alleles is optimal. Notably, as in Ref. (118), this estimate arises without any constraints from positive selection.

## Summary

This review has outlined how several relatively simple descriptions of single TCR–pMHC interactions have been used to understand aspects of TCR repertoire development. However, the discussion is necessarily incomplete. In particular, there is an extensive literature exploring the molecular mechanisms by which individual or collections of TCR discriminate between ligands of different affinities [see, for example, Ref. (100, 122–126)], which has direct relevance to thymic selection. It remains unclear how proximal TCR signals derived from multiple and diverse pMHC ligands can drive the emergence of specificity and MHC restriction in the post-selection repertoire, although the models of selection on ensembles of ligands have made steps in this direction (99, 121). Are repeated super-threshold contacts required for negative selection, or is a single encounter with a high affinity ligand sufficient to cause deletion?

Many of the models discussed here assume that a single interaction above a minimum signaling threshold is sufficient for positive selection. However, there is evidence that repeated or sustained TCR signaling is required during the DP stage for positive selection to occur [see, for example, Ref. (17, 127)]. This may explain findings that positive and negative selection take place concurrently (46).

Overall it is remarkable how much insight into the quantitative aspects of thymic selection has emerged from highly abstracted models. However, there remain a lot of open areas for research, and many of the questions raised in the introduction are still unresolved. Regulatory T cell development in particular has received very little attention from modelers, and already it appears that the simplest extension to the simple probabilistic fixed-threshold model to include a fixed range of affinity or avidity for Treg selection is not sufficient to explain many experimental observations (107). The task of synthesizing and reconciling the huge diversity of experimental data related to thymic development is a daunting one, but the information available is perhaps currently underexploited by theorists.

## Conflict of Interest Statement

The author declares that the research was conducted in the absence of any commercial or financial relationships that could be construed as a potential conflict of interest.
